# Effect of an artificial intelligence health education accurately linking system on self-management in young and middle-aged patients after percutaneous coronary intervention: a protocol

**DOI:** 10.3389/fcvm.2026.1879287

**Published:** 2026-09-30

**Authors:** Yueli Li, Mengke Shan, Machao Liu, Yannan Zhang, Meiqi Zhang, Pingping Qin, Xia Zhao, Yunhua Li

**Affiliations:** 1 Department of Cardiology, Xuanwu Hospital Capital Medical University, Beijing, China; 2 School of Nursing, Henan University of Science and Technology, Luoyang, Henan, China; 3 School of Education, Chengdu College of Arts and Sciences, Chengdu, China

**Keywords:** AI-HEALS, artificial intelligence, health management intervention, large language model, mobile health, post-PCI

## Abstract

**Background:**

Coronary artery disease is a common heart disease worldwide, and the morbidity and mortality rates among Chinese residents continue to rise. Percutaneous coronary intervention (PCI) can improve survival rates, but long-term management is required after the procedure. Traditional interventions have shortcomings such as dependence on medical institutions and the lack of personalized treatment plans. This study aims to evaluate the effectiveness of the Artificial Intelligence-based Health Education Precision Linkage System (AI-HEALS) in improving self-health management among young and middle-aged patients after PCI.

**Methods and analysis:**

This is a single-center, randomized, single-blind, parallel-group controlled trial. Eligible patients aged 18–59 years from Xuanwu Hospital, Capital Medical University, will be randomly allocated at a 1:1 ratio to receive either standard care plus the AI-HEALS intervention (intervention group) or standard care alone (control group). The primary outcome is the change in self-management ability from baseline to the 3-month intervention endpoint, assessed using the Coronary Heart Disease Self-Management Behavior Scale (CSMS). Secondary outcomes include medication adherence, dietary behavior, exercise frequency, and psychological indicators. Assuming an effect size of *d* = 0.69, 80% statistical power, and a two-sided significance level of *α* = 0.05, with an anticipated 20% dropout rate, the required sample size was calculated as 86 participants (43 per group). Primary analyses will follow the intention-to-treat principle. Generalized linear mixed models will be used for repeated measures outcomes, and multiple imputation by chained equations will handle missing data. Quantitative findings will be integrated with qualitative data from structured interviews conducted among intervention group participants.

**Ethics and dissemination:**

This study has been approved by the Ethics Committee of Xuanwu Hospital, Capital Medical University (Approval No.[2024] 409-002). Written informed consent will be obtained from all participants. Results will be disseminated through peer-reviewed publications and academic conferences.

**Clinical Trial Registration**: http://www.chictr.org.cn/, identifier ChiCTR2500100519.

## 1 Introduction

One of the most common heart diseases in the world is coronary heart disease (CHD), and the major cause of its development is myocardial ischemia caused by stenosis or occlusion of the coronary vessels (1). In China, the prevalence of coronary heart disease among the population aged 18 and above from 2020 to 2022 is estimated to be 758 cases of coronary heart disease per 100,000 people, including cases of acute myocardial infarction (AMI), patients who underwent percutaneous coronary intervention (PCI) and coronary artery bypass grafting (CABG), and patients who were hospitalized for unstable angina (2). In the past few years, the incidence and mortality of coronary heart disease in China have been increasing continuously. In China, the number of patients with coronary heart disease is estimated to be 11.39 million (3). It is worth noting that the age of patients with coronary heart disease is gradually decreasing, and the impact of coronary heart disease is no longer limited to the elderly but has spread to middle-aged and young individuals (3–5). Especially among the youth, the incidence of coronary heart disease has increased significantly (3–5). In China, the incidence rate of ischemic heart disease among the population aged 15–39 in 2021 is estimated to be 46.5 cases of ischemic heart disease per 100,000 people (3). In China, the number of patients aged 25–45 years with acute coronary events between 1990 and 2022 is estimated to have increased by 30% (3).

From a pathophysiological viewpoint, CHD is generally caused by atherosclerotic changes in the coronary arteries, which lead to stenosis or obstruction of the vessel lumen, thus resulting in compromised blood flow (6, 7). Stenosis in the coronary arteries compromises the supply of oxygen and nutrients to the heart muscle, thus resulting in myocardial ischemia. In extreme cases, this results in angina pectoris and myocardial infarction (8, 9). The atherosclerotic changes in the coronary arteries result from a multifactorial process involving changes in lipid metabolism, chronic inflammatory response, endothelial dysfunction, and blood pressure regulation (10, 11). There is evidence that hypertension, hypercholesterolemia, diabetes, smoking, and genetic factors are the primary risk factors for coronary artery disease (12–14). In addition, unhealthy lifestyle factors such as unhealthy eating habits, lack of exercise, and excessive psychological stress can accelerate this pathological process (15–17). In addition, myocardial ischemia results in severe pain in the chest region, breathing difficulties, and heart failure. In the long run, coronary artery disease can cause damage to the heart muscle, adversely affect quality of life, and increase susceptibility to heart attacks.

Percutaneous coronary intervention is a minimally invasive interventional procedure and is considered one of the major therapeutic options for the management of coronary heart disease (18, 19). Percutaneous coronary intervention can reopen occluded coronary vessels using catheter-based interventions and relieve myocardial ischemia through improved myocardial blood flow (18, 19). Annually, more than one million patients undergo percutaneous coronary interventions globally. With the increased incidence of coronary heart disease among middle-aged and young people, and the development of interventional procedures, the number of patients receiving percutaneous coronary intervention treatment is rising among the young population. In 2023, the number of patients who underwent PCI in China was 1.901 million. This number represents 22.7% of the patients with coronary heart disease who underwent in-hospital treatment (2). Among the patients with coronary heart disease who underwent interventional procedures, the most common form of interventional therapy is stent placement (68.8%), while the least common is non-drug-coated balloon angioplasty (5.7%) (2). It is worth noting that the number of patients aged 18–59 years receiving PCI is rising continuously. Although the survival rate of patients with coronary heart disease has improved among middle-aged and young patients receiving PCI, the long-term management of patients with coronary heart disease is vital in preventing in-stent restenosis and other cardiovascular complications (18–20).

At present, the long-term management strategies for patients after percutaneous coronary intervention mainly include drug therapy, lifestyle adjustment guidance, and regular medical follow-up (21, 22). Drug therapy mainly focuses on the use of antiplatelet drugs and lipid-lowering drugs to prevent restenosis and thrombosis (22, 23). Lifestyle guidance typically includes dietary adjustments, increased physical activity, smoking cessation, and psychological support (22, 24, 25). Regular medical follow-up enables clinical doctors to monitor patients' recovery status and adjust treatment plans accordingly (22). Although these traditional management methods are effective to some extent, there are still several obvious limitations. Firstly, traditional intervention measures largely rely on patients regularly visiting medical institutions, which may be inconvenient and economically burdensome for many people. Secondly, standardized intervention plans may not meet the personalized needs of patients, especially in terms of psychological support and behavior change promotion. In addition, maintaining and continuously implementing lifestyle changes in the patients' family and social environment can often be challenging. Finally, although traditional post-PCI management provides a certain degree of support, it often lacks personalized and real-time feedback mechanisms, which may not be sufficient to address the self-management challenges faced by young and middle-aged patients in their daily lives. Previous research has verified that negative illness perceptions are strong predictors of anxiety and depressive symptoms among CHD patients, and insufficient targeted psychological intervention in routine follow-up cannot correct maladaptive disease cognition, further impairing patients' long-term self-management ability (26). Therefore, more flexible and personalized management strategies need to be explored to enhance the self-management ability of this population and improve their long-term health status. Comprehensive and structured health education constitutes the first line of defense in the long-term management of patients after PCI. Many previous studies have shown that personalized health education provided before discharge and during home rehabilitation, especially when combined with mobile health technology and intelligent management platforms, can significantly improve patients' self-management abilities (27–29).

WeChat is a widely used social media platform in China. This platform is widely used by young and middle-aged people. This group has good digital literacy and is easily influenced by smart health technologies. Therefore, the system is named AI-HEALS and was developed by integrating the WeChat platform and artificial intelligence technology to provide a secondary prevention health education system for coronary heart disease. This system is designed based on the HAPA-MTM model, focusing on intelligence, structure, and personalization. This system aims to improve patients’ knowledge of disease-related issues, behavioral habits, and mental health, especially among young and middle-aged people who have undergone PCI.

The objectives of this study were:

1. To Evaluate the effectiveness of the AI-HEALS intervention in improving psychological indicators among young and middle-aged patients after PCI;
2. To evaluate the effectiveness of the AI-HEALS intervention in enhancing self-management abilities in young and middle-aged patients after PCI, with the primary endpoint assessed at the completion of the 3-month intervention and secondary endpoints assessed at 3, 6, and 9 months post-intervention;
3. To Evaluate participants' acceptability, usability, and satisfaction with the intervention program.

## 2 Method

### 2.1 Study design

A single-blind randomized controlled trial with a parallel group and mixed methods design was carried out in the Department of Cardiology of Xuanwu Hospital, Capital Medical University. This study aimed to investigate the effectiveness of the application of the AI-HEALS system based on the WeChat platform compared with traditional standard medical care. Patients were randomly assigned to two groups in a 1:1 ratio. In the control group, patients only received the standard medical care, such as routine examinations, follow-up, and education about the disease. In the intervention group, patients received the above-mentioned standard care plus the intervention of the AI-HEALS system, such as knowledge questions, indicator recording, reminders, and educational articles. To prevent cross-contamination between the two groups of patients, they were separated and stayed in different wards. The process of the study is shown in Figure 1.

**Figure 1:**
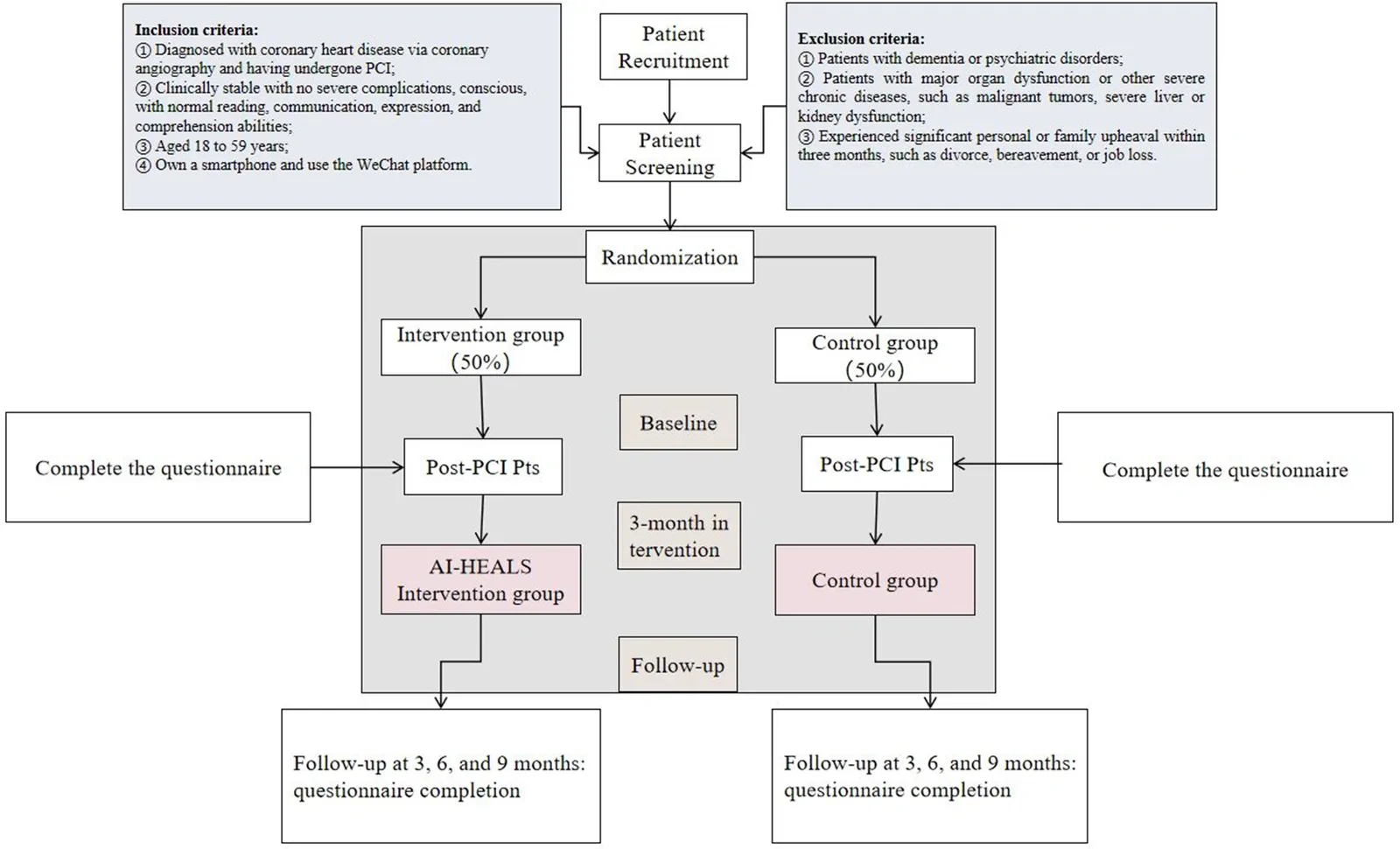
Patient recruitment and implementation flowchart.

### 2.2 Randomization and blinding

#### 2.2.1 Randomization

An independent biostatistician uninvolved in participant recruitment, enrollment, or intervention delivery generated the randomization sequence via IBM SPSS Statistics (version 24.0). Simple randomization with a 1:1 allocation ratio was adopted to allocate eligible participants to the intervention group or control group, without stratification or blocking. Each participant was assigned an exclusive personal identification code after randomization to guarantee consistent registration and data management throughout the trial. To avoid cross-contamination between groups, participants assigned to the intervention group were hospitalized in ward A, and control participants were hospitalized in ward B.

#### 2.2.2 Allocation concealment mechanism

To fully implement allocation concealment, sequentially numbered, opaque, sealed envelopes were used to store group allocation information. The independent biostatistician generated the random sequence, sealed each envelope in advance, and kept all envelopes under locked storage. When a participant completed screening and signed informed consent, the research coordinator opened the envelope in sequence to reveal the assigned group; recruiters had no access to the full random sequence or unused envelopes at any time.

#### 2.2.3 Blinding

Complete blinding of participants and intervention deliverers could not be achieved due to the inherent nature of the AI-HEALS intervention. Separate ward placement (Ward A for intervention, Ward B for control) was adopted to prevent cross-contamination, yet this one-to-one ward-group correspondence allowed clinical staff to deduce participants' group assignments, which may have trigger performance bias and detection bias. Multiple targeted measures were adopted to offset such risks:

1. Outcome assessors and data analysts remained fully blinded to group allocation throughout data collection and statistical analysis;
2. Identical standardized routine care protocols were implemented uniformly in both wards to eliminate distinguishable care differences;
3. A sensitivity analysis was planned to evaluate the potential confounding effect of response bias after all follow-up data were collected.

Unblinding was only authorized under medical emergency conditions, which required written records and could only be carried out by the principal investigator or formally designated study staff.

### 2.3 Study participants

#### 2.3.1 Inclusion criteria

1. Diagnosed with coronary heart disease via coronary angiography and having undergone PCI;
2. Clinically stable with no severe complications, conscious, with normal reading, communication, expression, and comprehension abilities;
3. Aged 18–59 years;
4. Willing to participate in this study
5. Own a smartphone and use the WeChat platform.

#### 2.3.2 Exclusion criteria

1. Patients with dementia or psychiatric disorders;
2. Patients with major organ dysfunction or other severe chronic diseases, such as malignant tumors, severe liver or kidney dysfunction;
3. Experienced significant personal or family upheaval within three months, such as divorce, bereavement, or job loss.

#### 2.3.3 Withdrawal criteria

1. Voluntary withdrawal from the study for personal reasons, such as scheduling conflicts or loss of interest;
2. Occurrence of a serious adverse event during the study requiring interruption of the intervention, such as severe physical illness or acute event (e.g., heart attack, severe infection).

### 2.4 Sample size

Sample size was calculated using G-Power 3.1.9.2. Based on a previous study (30), which evaluated a personalized cardiac rehabilitation education model on self-management in post-PCI patients using the same primary outcome measure (CSMS), the effect size was set at 0.69, power at 0.80, and alpha at 0.05, using a two-sided test. The calculated required sample size was 68 participants (34 per group). Estimating a dropout rate of 20%, the final sample size was determined as 86 participants (43 per group).

### 2.5 Participant recruitment

Participants in this study were young and middle-aged patients who underwent PCI surgery at a designated hospital and met the inclusion criteria. Medical staff first explained the study to the patients, and then researchers met with each patient face-to-face to explain the purpose of the study, the procedures, potential benefits, and potential risks in detail. After the patients fully understood the information, they were asked if they were willing to participate in the study; if they agreed, they signed an informed consent form on the spot and were enrolled.

### 2.6 Interventions

#### 2.6.1 Control group

The management of the control group will be carried out in accordance with the routine management guidelines of young and middle-aged patients after PCI. Routine management is conducted by doctors and a team of specialists. The patient will regularly visit their doctors to get check-ups and have their treatment plans adjusted according to their condition. The follow-up time is also fixed to monitor the dynamic changes in the patient's condition. In addition, routine examinations are conducted to monitor relevant clinical indicators. The patient also needs to attend health knowledge lectures to understand how to manage their diseases and change their lifestyle. If special needs are required, the patient will be referred to a specialist.

#### 2.6.2 Intervention group

In addition to routine care, the intervention group will also receive the AI-HEALS health system intervention. At the beginning of the study, baseline data will be collected first, including physical health indicators, psychological state assessments, and lifestyle characteristics. Follow-up appointments will be scheduled at discharge, and at three, six, and nine months after the intervention ended. The intervention team members come from diverse backgrounds, including public health professionals, clinicians, clinical nurses, psychologists, and statisticians. The specific interventions include the following aspects:

##### 2.6.2.1 Pre-deployment usability validation

Prior to formal trial initiation, the AI-HEALS system underwent a multi-stage usability validation process to ensure that the platform was intuitive, acceptable, and feasible for the target patient population. First, a structured heuristic evaluation was conducted by the multidisciplinary intervention team, including clinicians, nurses, public health experts, and technical developers. The evaluation focused on interface clarity, navigation logic, response readability, and task completion efficiency. Identified issues were iteratively refined before deployment. Second, a pilot test was conducted with 8 post-PCI patients representative of the target population. These patients were invited to use the AI-HEALS system and provided verbal feedback on their experience. This process allowed us to identify and address practical usability issues (e.g., login difficulties, navigation challenges, comprehension problems with health education content) before the formal trial began. During the formal trial, all intervention group participants receive personalized onboarding guidance from trained research staff, and a dedicated technical support channel is available throughout the intervention period.

##### 2.6.2.2 Workflow among patients, the AI system, clinicians, and researchers

The AI-HEALS intervention involves four key stakeholders with distinct roles throughout the trial. Patients interact with the AI-HEALS system via the WeChat Mini Program by asking questions through the KBQA system, receiving personalized responses, accessing standardized health education articles, setting reminders, and recording lifestyle data; they also attend scheduled follow-up visits for outcome assessments. The AI system processes patient inputs, generates responses using the Doubao Pro-32k large language model grounded in a curated knowledge base, and logs all interactions for review and quality monitoring. Clinicians provide routine clinical care to both groups, oversee patient safety, review flagged AI responses, and manage escalated clinical issues. The research team manages participant recruitment, enrollment, and randomization; conducts daily reviews of AI interaction logs and weekly independent quality reviews; and collects and analyzes outcome data. This four-stakeholder workflow ensures clear role delineation and maintains the integrity of intervention delivery, safety monitoring, and data collection.

##### 2.6.2.3 PCI postoperative knowledge-based question-answering system (KBQA)

The KBQA system provides patient-driven interactive responses based on retrieved clinical knowledge. This function does not rely on passive data collection and remains functional regardless of whether lifestyle records are available. To implement the knowledge-based Q&A function, we organized various health-related contents into a curated knowledge base, including disease characteristics, common causes, symptoms, treatment options, medication use, daily precautions, dietary recommendations, and appropriate physical activities. The knowledge base was independently maintained as an external document repository and integrated into the AI-HEALS system through a retrieval-augmented generation (RAG) framework. When patients submitted questions, the system retrieved the most relevant knowledge segments from the knowledge base and provided them as contextual information to the Doubao Pro-32k large language model to assist in generating evidence-based responses. The generated responses were subsequently delivered to patients through the WeChat platform. Following each query, the system provided three related recommended questions to facilitate further exploration of relevant health information and enhance patient education.

##### 2.6.2.4 Recording lifestyle factors and physiological indicators

The AI-HEALS platform can assist patients in recording their daily lives, such as weekly medication, dietary plans, and exercise. Patients can also input data directly into the system. Researchers can also directly view the content input by patients in the background. In this way, long-term tracking and monitoring of patients can be conducted. To ensure the accuracy of the data input by patients, the research team can work with the patients to develop the SMART plan before the commencement of the study. Data input by patients afterward can be used as a reference for the plan. Every Saturday, a reminder will be sent by the system to all patients to upload their data. In this way, the changes that the intervention has brought to patients' daily lives can be understood.

##### 2.6.2.5 System behavior in the absence of patient-input data

When patients do not submit self-management records, the AI-HEALS system does not make any inferences or assumptions about patient behavior. The reminder function continues to operate as configured by the patient, and health education articles continue to be delivered on schedule. No automated decisions or personalized recommendations are generated based on missing data. Researchers may follow up via WeChat or telephone to encourage recording, but no algorithmic action is triggered by the absence of data.

##### 2.6.2.6 Personalized reminder service

The AI-HEALS system provides a patient-configured reminder function, through which patients can set their own reminder times and types according to their individual needs (e.g., medication timing, exercise schedules). For example, patients can input a reminder to take their medication on time, exercise every day, and so on. After inputting a reminder into the system, a reminder message will be sent to the patients at a specific time. In this way, patients can improve their ability to comply with medical advice, develop healthy lifestyle habits, standardize their medication habits, and thus improve their self-management ability continuously.

##### 2.6.2.7 Automated personalized education articles for patients after PCI

The system broadcasts standardized health education articles to all intervention group participants on a fixed schedule—a short cardiovascular health tip daily and a post-PCI self-management article weekly. The article will cover advice on exercise, including choosing suitable types of exercise and activity intensity, and details regarding patient medication, such as how to administer the medication and what side effects to be aware of.

##### 2.6.2.8 Intervention duration and follow-up

The AI-HEALS intervention is delivered only during the 3-month active intervention period. After this period, all active intervention components (KBQA, reminders, and automated article) are discontinued. Participants in the intervention group receive no further active support from the system during the follow-up phase (3, 6, and 9 months post-intervention), receiving only routine clinical follow-up as per standard care. The follow-up period is designed to assess the sustainability of intervention effects.

##### 2.6.2.9 Adherence definition

In this study, adherence refers to platform adherence or intervention engagement, defined as the extent to which participants use and engage with the AI-HEALS intervention components. It does not directly indicate whether participants have fully implemented all health behavior recommendations in their daily lives. Actual behavioral changes will be evaluated using prespecified clinical and behavioral outcome measures. Platform adherence will be assessed using objective backend usage data from two primary dimensions: KBQA engagement and article engagement. For KBQA engagement, participants are expected to use the AI chatbot at least once per week over the 12-week intervention period. Therefore, the denominator for KBQA adherence is 12 expected weekly KBQA interactions. KBQA adherence will be calculated as the number of weeks in which the participant submitted at least one KBQA question divided by 12. For article engagement, the denominator will be the total number of health education articles pushed to the participant during the intervention period. Article engagement will be calculated as the number of effectively read articles divided by the number of articles pushed. Effective reading will be determined based on backend records, including article opening, reading duration, and reading completion status. Overall platform adherence will be calculated based on both KBQA adherence and article engagement adherence. Participants will be classified into three adherence levels: high adherence, defined as overall platform adherence ≥70%; moderate adherence, defined as 40%–69%; and low adherence, defined as <40%. In the per-protocol analysis, participants with overall platform adherence ≥70% will be considered to have adequately engaged with the intervention. In addition, backend engagement metrics—including total KBQA questions submitted, number of weeks with at least one KBQA interaction, number of effectively read articles, total article reading time, and effective reading rate—will be summarized descriptively to characterize participants’ engagement with the intervention.

## 3 Outcomes

### 3.1 Primary outcome

Changes in self-management abilities in young and middle-aged patients were recorded at baseline (pre-intervention), at the end of the 3-month intervention, and during follow-up at 3, 6, and 9 months post-intervention (31).

### 3.2 Secondary outcomes

Patient-reported outcomes and experience indicators for both control and intervention groups at the end of the 3-month intervention and at 3, 6, and 9 months post-intervention include changes in self-management behaviors and psychosocial aspects. Changes in Self-Management Behaviors: Dietary habits, physical exercise, medication adherence, smoking management, alcohol management, sleep management, quality of life, and activities of daily living. Changes in Psychosocial Aspects: Health literacy post-PCI, self-efficacy, depression, anxiety, stress, and social support.

### 3.3 Changes in self-management behaviors

Dietary habits, physical exercise, medication adherence, smoking management, alcohol management, sleep management, quality of life, and activities of daily living.

### 3.4 Changes in psychosocial aspects

Health literacy post-PCI, self-efficacy, depression, anxiety, stress, and social support.

Additionally, recruitment rates, dropout rates, and reasons for dropout will be collected. To ensure data homogeneity, demographic characteristics and study outcomes of dropouts and those with negative attitudes toward the trial will be compared during data analysis.

## 4 Variable measurement

Sociodemographic variables will be collected at baseline. Physical examinations and questionnaire surveys will be conducted at baseline, discharge, at the end of the intervention, and at 3, 6, and 9 months of post-intervention follow-ups (Table 1).

**Table 1:** Schedule of enrolment, interventions, and assessments.

Study period	Enrollment	Intervention	Follow-up	
Time Point	T_0_	T_1_	T_2_	T_3_
Eligibility Screening	√			
Informed Consent	√			
Allocation	√			
Sociodemographic Variables	√			
PSS-4	√	√	√	√
PHQ-4	√	√	√	√
PSSS-SF	√	√	√	√
NGSES-SF	√	√	√	√
CSMS	√	√	√	√
EQ-5D-5L	√	√	√	√
B-PSQI	√	√	√	√
SREBQ	√	√	√	√
IPAQ	√	√	√	√
ARMS-7	√	√	√	√
FCS-SF	√			
eHEALS	√			

T_0_ = 0-month baseline (pre-intervention), T_1_ = 3-month (end of intervention), T_2_ = 6-month follow-up, T_3_ = 9-month follow-up.

All questionnaires are Chinese validated versions.

PSS-4, 4-item perceived stress scale; PHQ-4, 4-item patient health questionnaire (depression and anxiety screening); PSSS-SF, perceived social support scale-short form; NGSES-SF, new general self-efficacy scale-short form; CSMS, coronary heart disease self-management behavior scale; EQ-5D-5L, EuroQol-5 dimensions 5 Levels; B-PSQI, brief Pittsburgh sleep quality index; SREBQ, self-regulation of eating behavior questionnaire; IPAQ-SF, international physical activity questionnaire-short form; ARMS-7, adherence to refills and medications scale; FCS-SF, family communication scale-short form; eHEALS, eHealth literacy scale.

### 4.1 Sociodemographic variables

A customized demographic questionnaire will be used to measure the following variables: sex, age, marital status, household registration type, average household size, education level, occupation, income, and detailed medical history.

### 4.2 Anthropometric variables

Height, weight, and waist circumference are measured using internationally certified instruments. To ensure accuracy, each indicator is measured twice. Height and weight are measured using calibrated, certified electronic devices. After measurement, the body mass index (BMI) is calculated by dividing weight (kg) by the square of height (m^2^). Waist circumference is measured at the navel using a soft measuring tape. After discharge, participants will be contacted via WeChat, telephone, or text message to confirm their participation status and obtain follow-up information. During routine communications, they will be updated on study progress, the resolution of any issues encountered, and the schedule for the next follow-up visit. The data is entered using an electronic data acquisition system, which has a high degree of standardization. To ensure the accuracy of the data entry, we have arranged for two people to enter the data. Sensitive information is first converted into a unified encoding format using a standard method like Unicode, and then encrypted. Only authorized researchers are allowed access to the data, and the data is backed up in real time to ensure that it is not lost.

## 5 Questionnaires

### 5.1 Self-management ability

Will be measured using the Coronary Heart Disease Self-Management Behavior Scale (CSMS). This scale includes three dimensions: daily life management, disease medical management, and emotional cognitive management. It uses a 5-point Likert scale. The sum of item scores in each dimension represents the dimension total, and the sum of dimension scores represents the total self-management behavior score. Higher scores indicate better self-management behavior. The scale has good reliability and validity, with a test–retest reliability of 0.910 and an internal consistency Cronbach's *α* coefficient of 0.913 (31).

### 5.2 The Chinese version of the 4-item perceived stress scale (PSS-4)

Is a widely validated brief self-report tool designed to assess an individual's perceived level of stress from life events over the past month (32). The scale focuses on evaluating stress triggers and the associated cognitive load, rather than direct emotional symptoms. It consists of 4 items, each rated on a 5-point Likert scale ranging from 0 (never) to 4 (very often), with total scores ranging from 0 to 16. Higher scores indicate greater perceived stress. The scale has demonstrated good reliability and validity in Chinese populations.

### 5.3 Depression and anxiety

Will be assessed using the abbreviated Patient Health Questionnaire-4 (PHQ-4), a brief self-report tool designed to assess symptoms of depression and anxiety over the past two weeks (33). The scale consists of four items, with the first two related to depression and the last two related to anxiety. Each item is scored on a 4-point scale: “Not at all” (0), “Several days” (1), “More than half the days” (2), and “Nearly every day” (3). The total score ranges from 0 to 12, with a Cronbach's *α* value of 0.833 (34).

### 5.4 The Chinese version of the perceived social support scale-short form (PSSS-SF)

Is designed to assess an individual's perception of social support (35). The scale consists of three items across three dimensions: family support (item 1), friend support (item 2), and other support (item 3). It uses a 7-point Likert scale ranging from 1 (very strongly disagree) to 7 (very strongly agree), with total scores ranging from 3 to 21. Higher scores indicate higher levels of perceived social support. The scale has good internal consistency, with a Cronbach's *α* of 0.84 (36).

### 5.5 The Chinese version of the new general self-efficacy scale-short form (NGSES-SF)

Is used to assess an individual's confidence and belief in their ability to complete tasks or cope with challenges (37). The scale includes three items across three dimensions: self-efficacy level or degree (item 1), strength (item 2), and generality (item 3), using a 5-point Likert scale ranging from 1 (strongly disagree) to 5 (strongly agree). Total scores range from 3 to 15, with higher scores reflecting higher levels of self-efficacy. The scale has good internal consistency, with a Cronbach's *α* of 0.876 (37).

### 5.6 The Chinese version of quality of life

Will be assessed using the EQ-5D-5L (EuroQol-5 Dimensions 5 Levels). The EQ-5D-5L includes five dimensions: mobility, self-care, usual activities, pain/discomfort, and anxiety/depression. Individuals select the appropriate level for each dimension based on their health status, forming a five-dimensional profile ranging from no problems to extreme problems (38). In addition to the descriptive system, the EQ-5D includes a VAS, allowing individuals to subjectively rate their current health on a scale from 0 to 100, providing a continuous variable for measuring overall quality of life. In seven studies involving cancer patients, the EQ-5D demonstrated high validity and reliability, with Cronbach's *α* values ranging from 0.72 to 0.90 (39–45).

### 5.7 The Chinese version of the international physical activity questionnaire short form (IPAQ-SF)

Consists of seven items designed to assess physical activity in adults aged 15–69 over the past seven days, including vigorous activity, moderate activity, and walking (46). Participants report the frequency and duration of each activity and weekday sitting time. Scoring involves converting activity duration into weekly MET-minutes (metabolic equivalent of task), with different activities assigned different MET values to quantify an individual's total physical activity level. This tool is widely used in public health research and has demonstrated acceptable reliability, with an intraclass correlation coefficient (ICC) of 0.79 and a coefficient of variation (CV) of 26% (47).

### 5.8 The Chinese version of the new brief version of the Pittsburgh sleep quality index (B-PSQI)

Is a condensed version used to measure individual sleep quality (48). The scale consists of 6 items across 5 dimensions, specifically measuring sleep efficiency (items 1 and 2), sleep onset time (item 3), sleep duration (item 4), sleep disturbances (item 5), and overall sleep quality (item 6). Bedtime and wake-up time are used to calculate sleep efficiency. Total scores range from 0 to 15, with higher scores indicating poorer sleep quality. The scale has acceptable internal consistency, with a Cronbach's *α* of 0.79 (48).

### 5.9 The Chinese version of the family communication scale-short form (FCS-SF)

Is used to measure communication abilities within the family system (36). The scale consists of 4 items across a single dimension, using a 5-point Likert scale from 1 (strongly disagree) to 5 (strongly agree). Total scores range from 5 to 20, with higher scores indicating higher levels of family communication. The scale has good internal consistency, with a Cronbach's *α* of 0.91 (49).

### 5.10 The Chinese version of the self-regulation of eating behavior questionnaire (SREBQ)

Is used to assess an individual's ability to regulate their eating behaviors (50). The scale consists of 5 items across a single dimension, using a 5-point Likert scale from 1 (never) to 5 (always). Total scores range from 5 to 25, with higher scores indicating a greater ability to regulate dietary habits. The scale has acceptable internal consistency, with a Cronbach's *α* of 0.75 (50).

### 5.11 The Chinese version of the eHealth literacy scale (eHEALS)

Aims to comprehensively assess an individual's knowledge, ease of use, and perceived skills in searching, evaluating, and applying electronic health information to solve real-world health problems (51). The scale includes 5 items across a single dimension, using a 5-point Likert scale from 1 (strongly disagree) to 5 (strongly agree). Total scores range from 5 to 25, with higher scores indicating higher levels of personal eHealth literacy. Its Cronbach's *α* is 0.913 (52), indicating good internal consistency.

### 5.12 The adherence to refills and medications scale (ARMS-7)

Is a 7-item self-report instrument designed to assess medication adherence among patients with chronic diseases, covering both medication-taking and refill behaviors, with total scores ranging from 7 to 28 (higher scores indicate poorer adherence) (53). The Turkish version of the ARMS-7 demonstrated good validity and reliability, with a content validity index of 0.94, a Cronbach's *α* of 0.75.

## 6 Statistical analysis

All statistical analyses will be conducted using IBM SPSS Statistics version 24.0 (IBM Corp., Armonk, NY, USA), Stata version 14.0 (StataCorp LLC, College Station, TX, USA), and Mplus version 7.4 (Muthén & Muthén, Los Angeles, CA, USA). Questionnaire data will be entered and managed in Microsoft Excel before being imported into the statistical software for analysis.

The primary analyses will follow the intention-to-treat (ITT) principle, including all randomized participants. Missing data will be assumed to be missing at random (MAR) and handled using multiple imputation by chained equations (MICE) with 20 imputed datasets, 50 iterations to ensure convergence, and predictive mean matching (PMM) with five nearest neighbors.

Baseline characteristics will be summarized using descriptive statistics. Continuous variables will be assessed for normality using the Kolmogorov–Smirnov test and presented as mean ± standard deviation (SD) or median [interquartile range (IQR)], as appropriate. Categorical variables will be presented as frequencies and percentages. Baseline group comparisons will be performed using the Student's *t*-test, Mann–Whitney *U* test, one-way analysis of variance (ANOVA), or the chi-square test, as appropriate.

The primary and secondary outcomes measured repeatedly over time will be analyzed using generalized linear mixed models (GLMMs), including fixed effects for group, time, and the group-by-time interaction, with participant-level random effects to account for within-subject correlations.

Exploratory analyses will be conducted to examine associations between behavioral patterns, mobile health application use, and study outcomes using linear regression, logistic regression, and structural equation modeling (SEM), as appropriate. In addition, the relationship between intervention adherence and outcomes will be examined using generalized linear models, with adherence treated as a continuous variable (defined as the total number of KBQA questions submitted during the 3-month intervention period) and changes in the primary outcome as the dependent variable, adjusting for baseline covariates. These analyses will assess whether greater engagement with the AI-driven question-and-answer system is associated with greater improvements in self-management ability.

Prespecified subgroup analyses will be performed according to participants' family communication levels and eHealth literacy to explore potential effect modification.

Sensitivity analyses will be conducted to assess the robustness of the findings. For the per-protocol analysis, participants in the intervention group with overall platform adherence ≥70% will be considered adherent to the intervention. In addition, backend engagement metrics—including total KBQA questions submitted, number of weeks with at least one KBQA interaction, number of effectively read articles, total article reading time, and effective reading rate—will be summarized descriptively to characterize intervention engagement.

All statistical tests will be two-sided, with *P* < 0.05 considered statistically significant.

Upon completion of the intervention and at 3, 6, and 9 months post-intervention, structured interviews will be conducted with participants from the intervention group to gather feedback on their experiences with the AI-HEALS program. The structured interview guide will consist of 8–10 open-ended questions covering four main areas: (1) perceived attractiveness and acceptability of the program, (2) usability and ease of engagement, (3) motivational and challenging factors for sustaining self-management behaviors, and (4) suggestions for further development of AI-HEALS. These interviews aim to provide deeper insights into the factors motivating participation and behavioral change. Participants will be invited to engage in approximately 30 min telephone or face-to-face in-depth interviews. Selection of participants will consider sociodemographic and clinical characteristics, including age, gender, education, income, marital status, time since diagnosis, number of stents implanted, presence of other chronic conditions, and living arrangements. Recruitment will continue until thematic saturation is achieved. Audio recordings of interviews will be transcribed verbatim, anonymized, and analyzed, with data coding and management conducted in NVivo (Version 12, QSR International, Doncaster, Australia). Both qualitative and quantitative data will be integrated to better interpret the results across multiple phases of the trial.

## 7 Study management

### 7.1 Data collection

The data statistical analysis plan is guided by statistical professionals. The chief researcher will regularly check the team's work progress and promptly rectify any issues such as incomplete surveys or inadequate follow-ups. If the questionnaire has design problems or the data is incorrectly recorded, data collection should be suspended and resumed only after rectification is completed. If significant adjustments are required to the content, all involved subjects will use the same modified version. The revised content will be reviewed by experts and a preliminary experiment will be completed to confirm that the content is standardized and the data is reliable before it is officially used.

Before the research officially begins, team members will communicate with the principal investigator weekly to ensure that all preparations proceed smoothly. Once the data collection phase begins, the principal investigator and the trial manager will communicate monthly to review the implementation of the plan and promptly identify and resolve any communication barriers that may arise during the research.

Before the research begins, professionals will provide systematic training to the team, ensuring that each member is familiar with the research steps and related technical operations. Each participant was assigned a unique number and had an independent data file. The assessment time was uniformly arranged, and a mature questionnaire that had been tested for reliability and validity was used for the assessment to ensure that the data of the intervention group and the control group had a basis for comparison. When data anomalies are found, they need to be verified against the original questionnaire. Subsequent processing can only proceed after the data is confirmed to be accurate.

### 7.2 Data storage and archiving

We will archive all study data, including participant ID codes, original records, research documents, and correspondence, in a dedicated research database. After study completion, all raw data and related documents will be stored in line with legal and regulatory requirements to ensure long-term preservation and traceability.

All collected information will be entered into a secure, encrypted computer system. Only researchers approved for this project will have access to the data during the study period. In accordance with relevant regulations, all data will be safely stored after the study ends and then destroyed appropriately as required.

### 7.3 Ethics committee

The data monitoring committee members were selected from the ethics committee of Xuanwu Hospital, with a minimum of two members, neither of whom had a conflict of interest with this study. The committee oversees the daily conduct of the research and can directly report any issues to the ethics committee. Its work is independent of the principal investigator's management. When research deviates significantly from the plan, or when the plan is adjusted without following proper procedures, the committee may suspend the research process, and in serious cases, terminate the project directly. The ethics committee will also conduct a comprehensive review of the overall progress of the research every six months.

If any adverse event occurs during the study, the team must report it to the ethics committee within 24 h. For minor or moderate adverse events that do not pose an immediate safety threat, the participant may continue in the trial after appropriate clinical assessment and management, with close monitoring as determined by the clinical team. For severe or life-threatening adverse events, the participant must be withdrawn from the trial, and the event will be managed according to standard clinical protocols. All adverse events and the decisions regarding continuation or withdrawal will be documented and reported to the ethics committee. If the AI system experiences a serious program error or obvious malfunction during use, the experiment must be immediately suspended. The research can only be resumed after technical debugging and interface optimization have been completed and verified to be correct, to ensure the safety of the subjects and the reliability of the research data.

No compensation is offered for trial-related injuries, as the intervention involves only non-invasive health education delivered via a WeChat Mini Program and does not include any experimental drug, device, or invasive procedure. Participants experiencing any adverse events or health concerns during the trial are advised to seek medical care through routine clinical channels. This is consistent with the informed consent form, which states that no compensation is provided.

### 7.4 Informed consent

The informed consent process will be transparent and open, and will strictly follow applicable ethical guidelines. A detailed written informed consent form will be provided to young and middle-aged patients with coronary heart disease who have undergone PCI and meet the study eligibility criteria. The consent form will include information about the study purpose, specific procedures, potential benefits and risks, data confidentiality and disclosure measures, as well as other relevant aspects such as risk management, data storage, and security.

All collected data will be entered into an encrypted computer system, and access during the study will be restricted to authorized research personnel. After study completion, data will be securely stored and destroyed in accordance with relevant regulations.

Patients will be given sufficient time to read the consent form, ask any questions needed to understand their participation, and make their own voluntary decision. Researchers will clearly inform participants that they may withdraw from the study at any time without any negative consequences. Participants will also receive contact information for the research team so they can ask further questions or seek clarification in the future. If any new events or information arise that could affect participants' willingness to continue, researchers will promptly inform participants of any changes to the informed consent process.

Participants assigned to the intervention group who later decide to withdraw will not be penalised. Those who withdraw during the intervention phase will not be reassigned to the control group. In accordance with the intention-to-treat principle, all randomised participants will be retained in their original groups for the primary analysis, and withdrawal will not lead to exclusion from the final analysis. When feasible, the research team will conduct structured interviews with withdrawing participants to explore their reasons for discontinuation. In addition, through an ethical informed consent process, participants will be made aware of the follow-up communication and support available after withdrawal, ensuring they fully understand their rights and all possible avenues within the study.

The AI-HEALS system used in this study is technically supported by an external commercial large language model (ByteDance Doubao Pro-32k API) for its question-and-answer function. Free-text queries entered by participants into the system are transmitted to this external API via TLS-encrypted protocol to generate real-time responses.

All data are de-identified prior to transmission (i.e., direct identifiers such as name and hospital admission number are removed), and industry-standard encryption measures are applied during transmission. The external API provider processes the query text solely for response generation and does not retain, store, or record any patient data, nor is it permitted to use the data for model training or any other purposes. All interaction logs are ultimately stored only on the hospital-internal server designated by the research team.

### 7.5 Strategies to improve intervention adherence

To improve patient cooperation, several strategies will be implemented. First, during the recruitment and informed consent signing stage, the responsibilities and specific requirements for patients during the observation period will be clearly explained, while also focusing on establishing a trusting relationship with patients. Throughout the research process, patients will be continuously provided with education and consultation related to disease management and prevention. The characteristics of the disease itself, the importance of treatment adherence, and how to prevent possible complications will be explained to patients one by one. In addition, the research team will summarize the interactions between patients in the intervention group and the AI-HEALS system every week. If a decline in participation is identified, timely adjustments will be made to ensure that patients can continue to participate in the study.

### 7.6 Study results dissemination plan

The research team will prepare a summary of the RCT results and send it via email to individuals who indicated on the informed consent form that they wished to receive results. Study results will be disseminated through publications in domestic and international peer-reviewed journals and presentations at relevant academic conferences. Upon request from other hospitals, study results will be provided to their medical staff, provided that such requests are reasonable and comply with applicable regulations.

### 7.7 Overview of the artificial intelligence system

This study used the “Doubao Pro-32k” large language model through an API-based integration. The model itself was not retrained or fine-tuned for this study. Instead, AI-HEALS employed a retrieval-augmented generation (RAG) framework to combine the general language capabilities of the large language model with a curated clinical knowledge base.

The knowledge base was maintained as an external repository and consisted of 5 clinical guidelines, 112 patient-oriented educational articles, and 96 frequently asked question-and-answer pairs, with a total plain-text size of approximately 32.0 MB. The knowledge sources included clinical guidelines, expert consensus documents, and educational materials related to coronary heart disease management, medication adherence, lifestyle modification, diet, physical activity, warning signs, and psychological support. The knowledge base has incorporated relevant chapters on coronary heart disease from two authoritative Chinese textbooks in clinical medicine and nursing, which serve as core sources for post-PCI secondary prevention content: (1) Ge JB et al., Internal Medicine (People's Medical Publishing House, 2024), Chapter 4 of the Circulatory System, which covers antiplatelet therapy, lipid management, blood pressure control, and rehabilitation management after PCI; and (2) You LM, Wu Y., Medical Nursing (7th Edition, People's Medical Publishing House, 2022), Chapter 3 of the Circulatory System, which covers nursing assessment, medication guidance, wound care, and health education for post-PCI patients. These textbook materials have been reviewed by clinical experts and transformed into patient-friendly educational articles and Q&A pairs, which underpin the generation of PCI-related responses. Nevertheless, we fully agree that the knowledge base would benefit from more explicit authoritative sources specifically for PCI secondary prevention. Therefore, we plan to include the latest Chinese guidelines on post-PCI secondary prevention in the next knowledge base update.

All educational materials were rewritten into patient-friendly language by the research team. Before inclusion in the knowledge base, each item was independently reviewed by at least two clinical team members (cardiologists or cardiac rehabilitation nurses) to ensure clinical accuracy, consistency with guidelines, readability, and avoidance of misleading recommendations.

During each patient query, the system retrieves the top 3–5 most relevant knowledge segments from the database (typically approximately 4,000–8,000 tokens) and provides these retrieved contents together with the patient's question as contextual information within the 32k context window of the Doubao Pro-32k model. The 32k context window is therefore used for processing retrieved evidence rather than storing the entire knowledge base.

This RAG-based design allows the knowledge base to be updated independently without modifying the underlying language model, improves transparency by linking generated responses to source documents, and reduces the risk of unsupported AI-generated information.

Knowledge base updates are performed when new clinical guidelines become available or when patient interactions reveal knowledge gaps. All updates are recorded using version numbers, update dates, and approval records to maintain traceability. When tested on the FlagEval benchmarking platform, Doubao Pro-32k achieved a total score of 77.75, slightly higher than GPT-4o's 73.51, with a particularly strong performance in knowledge application (91.14 vs. 86.71), which is critical for generating evidence-based medical information.

The Doubao Pro-32k model was selected based on its demonstrated advantages in Chinese medical contexts, as supported by peer-reviewed literature. A 2025 study published in *Scientific Reports* evaluated Chinese large language models across the full clinical workflow using simulated patient cases, showing that Doubao performed comparably to ChatGPT-4 across all dimensions and outperformed human emergency physicians in diagnostic reasoning and patient management (54). These findings support that Doubao is particularly well-suited for Chinese-language medical applications. In addition, ByteDance provides enterprise-grade API services with TLS-encrypted transmission and role-based access control, ensuring full compliance with China's healthcare data protection regulations. Patient data remain within China's borders and are not used for model training. The model also offers optimized integration with the WeChat platform, which is essential given that the AI-HEALS intervention is delivered through a WeChat-based Mini Program.

To ensure consistency of AI-generated responses throughout the trial, the system is configured to call a fixed, specified version of the Doubao Pro-32k model for the entire trial period. The API version is explicitly specified in each request, and the version used for each response is logged in the backend system. Updates are permitted only under predefined conditions and will be documented as protocol amendments.

Prior to trial initiation, the AI-HEALS system was validated using a set of post-PCI clinical questions, with responses reviewed by independent clinical experts to confirm acceptable accuracy and safety. During the trial, a continuous human monitoring protocol is in place. All AI-generated responses are logged in real time. Trained researchers review every interaction log daily to identify potentially inaccurate, incomplete, or unsafe responses. In addition, two independent clinical experts review a random sample of 20% of responses weekly, following a standardized rubric covering clinical accuracy, completeness, safety, and clarity. Inter-rater reliability is assessed using Cohen's Kappa (*κ* ≥ 0.80), with disagreements resolved by a third senior clinician. Identified errors are recorded and used for weekly system updates. All review records are documented to ensure transparency and auditability. The AI-HEALS system was implemented using the commercial Doubao Pro-32k API (ByteDance) and a custom-built knowledge base. Doubao Pro-32k is a commercial large language model accessed via API, and its internal source code is not publicly available. Therefore, no model code can be provided. No custom AI model code was developed for this study. The knowledge base content (clinical guidelines, educational articles, and Q&A pairs) and the RAG pipeline configuration are available from the corresponding author upon reasonable request. The overall system architecture, illustrating the workflow from patient input through the WeChat Mini Program, RAG-based retrieval and response generation, knowledge base management, and human review layers, is presented in Figure 2.

**Figure 2:**
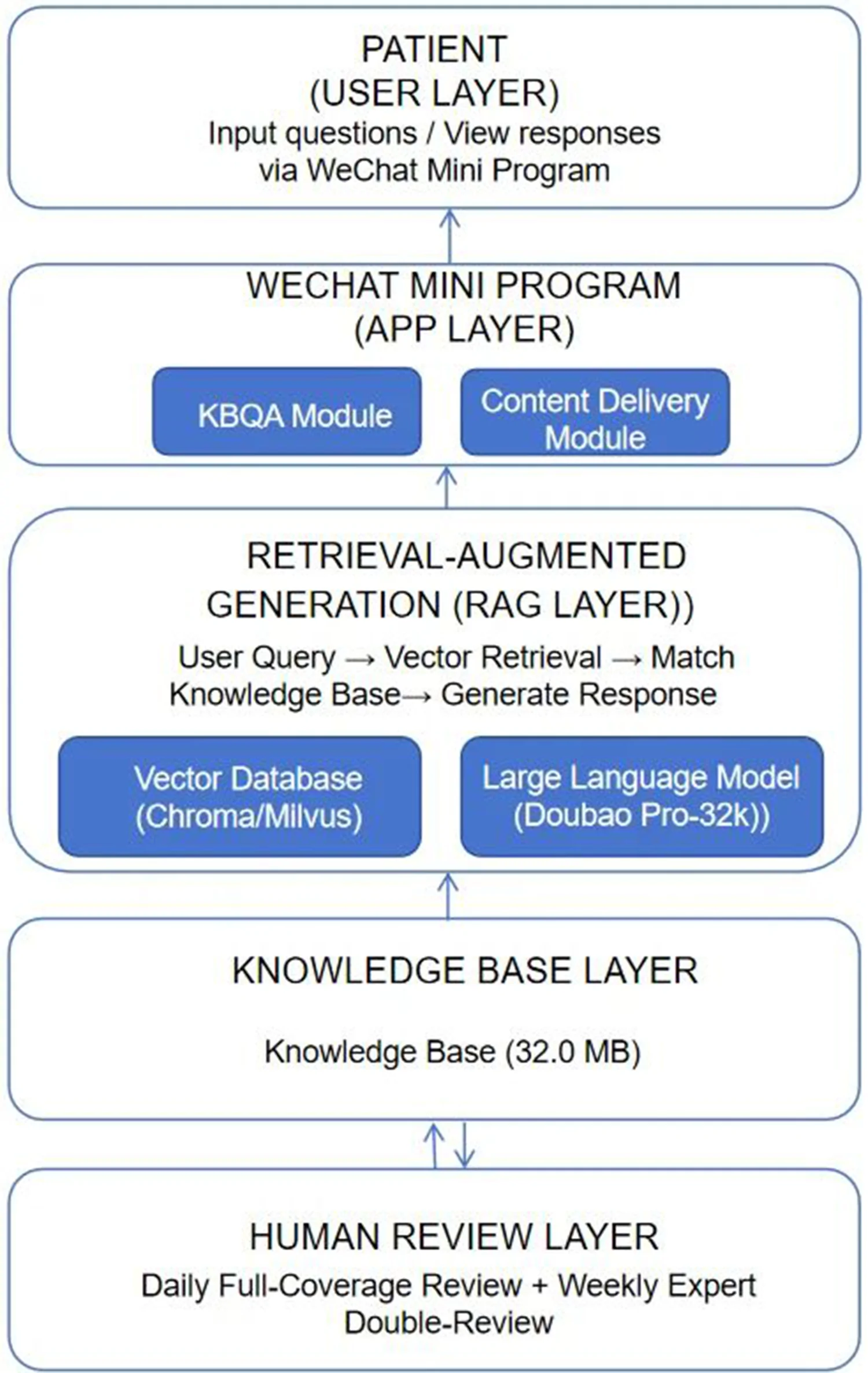
System architecture of the AI-HEALS platform.

### 7.8 Safety and quality control mechanisms

In this study, the AI-HEALS system is positioned primarily as a health education and self-management support tool, rather than an emergency symptom recognition, clinical diagnosis, treatment decision-making, medication adjustment, or psychological crisis intervention system. The current version of the system does not yet have the capability to implement real-time keyword-triggered alerts or automated escalation to the clinical team. To mitigate potential risks, the research team has implemented safety management measures including patient enrollment training, fixed platform safety disclaimers, AI Q&A safety statements, daily backend log reviews, and, when necessary, manual referral to the clinical team. Patients are explicitly instructed that for high-risk situations—such as chest pain, severe shortness of breath, syncope, rapid clinical deterioration, suspected severe adverse drug reactions, unauthorized discontinuation of essential medications, or self-harm/suicidal ideation—they should not rely on AI-generated responses, but should immediately contact their attending physician, visit the nearest emergency department, or call the local emergency hotline.

Prior to formal patient use of the AI-HEALS system, the research team will conduct a pre-deployment validation test to evaluate the accuracy, safety, and appropriateness of AI-generated responses. The test questions will be developed jointly by the research team and clinical experts, covering common health education and self-management scenarios encountered by post-PCI patients, including post-operative symptom recognition, management of chest pain or chest tightness, bleeding risk assessment, antiplatelet medication adherence, medication safety, blood pressure and lipid management, diet and exercise, follow-up visits, psychological support, and emergency situations. Each AI response will be independently evaluated by two clinical experts with relevant experience, who will assess the responses based on accuracy, completeness, safety, and appropriateness. Inter-rater reliability will be assessed using the Kappa coefficient (*κ*), with *κ* ≥ 0.80 considered acceptable.

If the Kappa coefficient falls below 0.80, the research team will suspend formal deployment of the system or temporarily suspend the relevant module. A third senior clinician will arbitrate the disagreements, and the research team will revise the knowledge base content, prompt settings, and AI response boundaries based on expert feedback. Re-testing will be conducted after revisions, and the AI Q&A module will only be deployed for patient use when the re-test results meet the pre-specified reliability and safety standards.

During system operation, the research team will continue daily backend log reviews and has established suspension criteria for the AI Q&A module. Critical errors include, but are not limited to: recommending that patients discontinue or adjust antiplatelet agents, antihypertensives, or lipid-lowering medications on their own; failing to provide immediate medical attention alerts for high-risk symptoms such as chest pain, severe chest tightness, dyspnea, syncope, major bleeding, or melena; providing recommendations that could delay treatment or increase patient risk; and failing to appropriately address psychological crisis content such as self-harm or suicidal ideation. Should any single critical error occur, the relevant AI Q&A function will be suspended immediately, and clinical experts and technical staff will jointly review, revise, and re-test the system. For non-critical but repeated errors—defined as inaccurate, incomplete, or inappropriate responses to the same type of question occurring three or more times within one week, or when the proportion of inaccurate/inappropriate responses exceeds 5% during routine spot checks—the relevant module will also be suspended, reviewed, and revised. The function will only be restored after revisions are completed and confirmed by expert review.

## 8 Discussion

This study aimed to evaluate the effect of AI-HEALS intervention on self-management ability in young and middle-aged patients after PCI through a randomized controlled trial. The primary outcome was changes in self-management ability, and secondary outcomes included medication adherence, dietary behavior, exercise frequency, and psychological indicators (depression, anxiety, self-efficacy, etc.). AI-HEALS integrates large language model question answering, behavior recording, personalized reminders, and health education article delivery, and the intervention content is designed based on the MTM-HAPA theoretical framework.

In recent years, several systematic reviews have confirmed that mobile health interventions can improve self-management and clinical outcomes in patients after PCI. Li et al. conducted a systematic review and meta-analysis, including four RCTs published between January 2020 and April 2025, involving a total of 827 patients after MI/PCI. The results showed that app-based intervention was significantly associated with a reduced risk of unplanned readmission (RR: 0.45, 95% CI: 0.23–0.89, *P* = 0.0219), with more significant benefits in studies with higher adherence and longer follow-up periods (55). The iCARE system RCT by Hou et al. confirmed that personalized intelligent intervention significantly improved adherence to healthy eating and medication at 3, 6, and 12 months (56). Young Chinese people generally accept health education delivered via social media digital platforms (57). Besides, digital psychological intervention tools can effectively adjust patients' unhealthy cognition (58).

The theoretical innovation of AI-HEALS lies in integrating MTM and HAPA, covering the entire chain of “cognitive restructuring–behavioral activation–habit consolidation.” MTM, as a fourth-generation theory of health behavior change, defines independent determinants for both the activation and maintenance stages, compensating for the lack of phased design in traditional theories (59).

This study has several limitations. First, as a single-center randomized controlled trial, the findings may have limited generalizability to other healthcare settings and populations. In addition, the sample size was calculated based on the effect size reported in a single previous study, which may have overestimated the intervention effect; therefore, the findings should be interpreted with caution. Second, because of the nature of the intervention, blinding was not feasible, and participants in the intervention group received more frequent contact than those in the control group, which may have introduced attention-related bias. Third, although participants were followed for 12 months, this period may be insufficient to evaluate the long-term sustainability of behavioral changes and clinical outcomes. Finally, the personalized component of AI-HEALS mainly relied on participants' active use of the knowledge-based question-answering (KBQA) system, and the large language model has not yet been integrated with real-time behavioral or physiological data, limiting the delivery of fully adaptive personalized support. Future multicenter trials with larger sample sizes, longer follow-up, and more advanced AI-driven personalization are warranted to validate and extend these findings.

## 9 Trial status

The study began in April 2025 and is expected to end in August 2026. A total of 86 participants have been enrolled (43 per group), and recruitment has been completed. Follow-up assessments are ongoing as scheduled. The study protocol (Version 1.0) was registered with the Chinese Clinical Trial Registry on April 10, 2025 (ChiCTR2500100519).

On 28 September 2025, the core research team made a collective decision to formally remove physiological indicators (including blood pressure, lipid profiles, and fasting blood glucose at 3, 6, and 9 months post-intervention) from the secondary outcome measures. This decision was based on two considerations: (1) the study lacked central laboratory quality control, and some participants were unable to return to the study centre hospital for follow-up assessments, which would have resulted in physiological data derived from multiple sources with inconsistent testing conditions, leading to substantial heterogeneity that could compromise accuracy and comparability; (2) these physiological indicators are significantly influenced by medication adherence and pre-testing conditions, and thus cannot directly or sensitively reflect the effects of a behavioural intervention centred on health education, rendering their inclusion as secondary outcomes scientifically unjustified. Importantly, this decision was made prior to any review or analysis of outcome data: the first intervention-group participant was enrolled on 10 July 2025, and the first scheduled 3-month post-discharge follow-up (the first time point for collecting the aforementioned physiological indicators) occurred on 20 October 2025. The decision therefore predated the generation of any follow-up physiological data, effectively avoiding the risk of selective reporting bias.

A formal protocol amendment (Version 2.0) was submitted to the Hospital Ethics Committee on 1 November 2025 and is currently under review (the approval letter is pending due to the rigorous ethical review process). Once ethical approval is obtained, the updated protocol will be promptly registered on the ChiCTR platform.
